# Nasal saline irrigation with azelastine-fluticasone nasal spray in moderate-to-severe persistent allergic rhinitis: a randomized controlled trial

**DOI:** 10.3389/falgy.2025.1622510

**Published:** 2025-10-13

**Authors:** Song Li, Rui Xu, Shaoqing Yu, Min Wang, Jiangang Fan, Ming Chen, Xiaoyang Gong, Qingjia Gu, Fenghong Chen, Ling Jin, Congli Geng, Maoxiao Yan, Changyu Qiu, Meiping Lu, Lei Cheng

**Affiliations:** 1 Department of Otorhinolaryngology, The First Affiliated Hospital with Nanjing Medical University, Nanjing, China; 2 Allergy Department, Otorhinolaryngology Hospital of the First Affiliated Hospital of Sun Yat-sen University, Guangzhou, China; 3 Department of Otolaryngology-Head and Neck Surgery, Tongji Hospital, School of Medicine, Tongji University, Shanghai, China; 4 Department of Otorhinolaryngology-Head and Neck Surgery, Peking University People’s Hospital, Beijing, China; 5 Department of Otolaryngology-Head and Neck Surgery, Sichuan Provincial People’s Hospital, University of Electronic Science and Technology of China, Chengdu, China; 6 Department of Otolaryngology, The Second Affiliated Hospital, Zhejiang University School of Medicine, Hangzhou, China; 7 Department of Otorhinolaryngology, The Second Affiliated Hospital of Guangzhou Medical University, Guangzhou, China; 8 Department of Allergology & Clinical Allergy Center, The First Affiliated Hospital with Nanjing Medical University, Nanjing, China

**Keywords:** allergic rhinitis, intranasal corticosteroids, intranasal antihistamines, nasal saline irrigation, randomized controlled trial

## Abstract

**Background:**

Symptom control in patients with moderate-to-severe persistent allergic rhinitis (PAR) who remain inadequately controlled on intranasal corticosteroid monotherapy remains challenging, highlighting the urgent need for more effective treatments. This study aimed to determine whether the addition of nasal saline irrigation to a regimen of intranasal corticosteroids and antihistamines can further improve symptoms in patients with moderate-to-severe PAR.

**Methods:**

A multicenter, randomized, open-label, controlled trial was conducted, enrolling 248 eligible patients aged 12 years and above from six clinical centers. They were randomized 1:1 into two groups. The experimental group received nasal saline irrigation combined with azelastine-fluticasone (Aze-Flu) nasal spray, and the control group was treated with azelastine nasal spray and fluticasone nasal spray. The primary outcome was the least-squares-mean (LSmean) change in total nasal symptom score (TNSS) from baseline to four weeks, with secondary outcomes including LSmean change in TNSS from baseline to two weeks, subscores, rhinoscopic scores, visual analogue scale (VAS), and rhinoconjunctivitis quality of life questionnaire (RQLQ) scores.

**Results:**

Both groups exhibited significant reductions in TNSSs from baseline (*p* < 0.001). In comparison to the control group, the experimental group exhibited greater LSmean changes in TNSS scores following either two or four weeks of treatment (*p* < 0.001 at both time points). The experimental group presented more favorable changes in rhinoscopy scores, VAS scores, and RQLQ scores. Both groups showed no substantial differences in adverse events, indicating a comparable safety profile.

**Conclusion:**

Nasal saline irrigation combined with Aze-Flu nasal spray provides additional benefits in managing moderate-to-severe PAR, with good safety and tolerability. This combination therapy could be a valuable option in primary care settings.

**Clinical Trial Registration:**

## 1 Introduction

Allergic rhinitis (AR), a non-infectious chronic inflammatory disease of the nasal mucosa in atopic individuals exposed to allergens, is primarily mediated by immunoglobulin E (IgE). The White Book on Allergy published by the World Allergy Organization (WAO) states that the prevalence of AR has risen to 10%–30% in adults and 40% in children (1). Its prevention and treatment also pose significant challenges to general practitioners. As the most prominent chronic inflammatory diseases of the upper respiratory tract, AR exerts serious impacts on patients' quality of life and a heavy burden on their socioeconomic status (2–4). The annual medical cost per patient for managing AR in Beijing, China, is up to 195.6 euros, and the national annual socioeconomic cost may reach 440.9 million euros (5). In the United States, the direct annual economic cost due to AR exceeds $4.5 billion (6). Furthermore, AR decreases labor efficiency, leading to an economic loss of approximately 30–50 billion euros per year across European Union countries (7). These data underscore the profound benefits of an effective AR therapy on both the healthcare and economy of one country.

In patients with moderate-to-severe persistent allergic rhinitis (PAR) who continue to experience symptoms despite guideline-recommended first-line intranasal corticosteroid (INCS) monotherapy (8), current evidence supports the addition of intranasal antihistamines (INAH) as a step-up combination approach (2, 9). Compared to INCS alone, INCS combined with INAH provides more clinical benefits to patients with moderate-to-severe PAR patients (10–15). Nevertheless, a significant number of patients with moderate-to-severe PAR still struggle with inadequate symptom control (8). Nasal saline irrigation is hypothesized to function by reducing nasal congestion, facilitating the elimination of secretions, and enhancing the local bioavailability of nasal medications, thereby improving the efficacy of nasal medications and alleviating nasal symptoms (16–19). It is widely used in the primary care of AR. The question arises whether incorporating nasal saline irrigation into INCS combined with INAH might further enhance symptom relief in patients with moderate-to-severe PAR. While regional studies on the use of nasal saline irrigation for AR have indicated its potential benefits in managing the condition (20–23), the evidence from these studies is considered to be of low or very low quality (17). Consequently, there is a lack of robust, large-scale clinical evidence to firmly support the use of nasal saline irrigation as an adjunct treatment in primary care for moderate-to-severe PAR. Therefore, we designed this multicenter, randomized, controlled, open-label, controlled trial across six centers nationwide.

## 2 Materials and methods

### 2.1 Setting, design, and participants

This study was a prospective, randomized, open-label, controlled clinical trial conducted across six centers in China from January 2023 to December 2023. The trial was designed to assess the efficacy and safety of combining nasal saline irrigation with azelastine-fluticasone (Aze-Flu) nasal spray in managing moderate-to-severe PAR. A total of 248 subjects who met the inclusion and exclusion criteria were enrolled and randomly assigned to the experimental group and control group in a 1:1 ratio. The experimental group received nasal saline irrigation combined with Aze-Flu nasal spray, and the control group was treated with azelastine hydrochloride nasal spray and fluticasone propionate nasal spray. The inclusion and exclusion criteria are detailed in the Supplementary Material S1.

The study was conducted across six clinical centers in China, including the First Affiliated Hospital with Nanjing Medical University, the First Affiliated Hospital of Sun Yat-sen University, Tongji Hospital of Tongji University, Peking University People's Hospital, Sichuan Provincial People's Hospital, and the Second Affiliated Hospital of Zhejiang University School of Medicine. Using block randomization, at least 20 patients (totaling 128) were enrolled from each center involved in this study (the First Affiliated Hospital with Nanjing Medical University was assigned 28 block randomizations, while each of the other five medical centers was assigned 20). The remaining 120 patients were included using a central randomization with a competitive enrollment approach. Random numbers were generated to allocate the enrolled patients into the experimental group and the control group. Enrollment encompassed screening, washout period, and a 4-week treatment. Additional details regarding screening and washout period are shown in Supplementary Material S2. The pharmacological regimens and irrigation solutions used in this study are detailed below.

Experimental arm: Aze-Flu fixed-dose combination nasal spray (CF PharmTech, Inc., Suzhou, Jiangsu, China). Each actuation delivers 137 µg azelastine hydrochloride and 50 µg fluticasone propionate; 120 sprays per bottle. One spray per nostril, twice daily (morning and evening).

Control arm: sequential administration of azelastine hydrochloride nasal spray (Guizhou Yunfeng Pharma Co., Ltd., Xingyi, Guizhou, China). Each actuation delivers 70 µg azelastine hydrochloride; 140 sprays per bottle. Two sprays per nostril, twice daily. Fluticasone propionate nasal spray (GlaxoSmithKline, S.A., Madrid, Spain). Each actuation delivers 50 µg fluticasone propionate; 120 sprays per bottle. One spray per nostril, twice daily, administered 15 min after the azelastine spray.

Nasal irrigation solutions: Weeks 1–2: hypertonic saline nasal irrigation (CF PharmTech, Inc., Suzhou, Jiangsu, China), 2.3% NaCl, 15 ml per nostril, twice daily. Weeks 3–4: normal saline nasal irrigation (CF PharmTech, Inc., Suzhou, Jiangsu, China), 0.9% NaCl, 15 ml per nostril, twice daily.

This design was deliberately adopted so that, from the patient's perspective, both arms entailed an identical regimen of two intranasal medications, thereby minimizing psychological bias associated with the added saline irrigation in the experimental arm. The detailed intervention protocols are described in the Supplementary Material S2.

### 2.2 Outcome measures

The participants were strictly followed up, as described in Supplementary Material S3. The changes in the scores of parameters subsequent to treatments, in contrast to baseline scores, were measured to evaluate efficacy outcomes. These changes were computed using the least squares mean (LSmean) based on post-treatment and pre-treatment scores. A negative value indicated a reduction in symptom scores, and a larger absolute negative value indicated a better outcome.

The primary outcome was the LSmean of the total nasal symptom score (TNSS) after four weeks of treatment. The TNSS evaluates a range of nasal symptoms, encompassing congestion, itching, rhinorrhea, and sneezing. Each symptom is scored on a scale from 0 to 3, with a higher score indicating a more serious symptom, and the scores of all symptoms are summed up as TNSS ranging from a minimum of 0 to a maximum of 12.

The secondary outcomes included: (1) The LSmean of the TNSS after two weeks of treatment. (2) The LSmean of the subscore in TNSS after two and four weeks of treatment. (3) LSmean change in total rhinoscopy score at 4 weeks, assessed by physicians blinded to group allocation and to patient-reported symptom scores (methodological details in Supplementary Material S4). The total rhinoscopy score is a sum of scores for four distinct items, each ranging from 0 to 3. Consequently, a total rhinoscopy score of 0–12 is calculated to reflect the nasal conditions. (4) The LSmean of daily visual analogue scale (VAS) total scores during the first two weeks of treatment. The VAS total score is the sum of the scores for the four nasal symptoms: congestion, itching, rhinorrhea, and sneezing, with each symptom rated on a scale of 0–10. Thus, the range of a VAS total score is 0–40. (5) The LSmean of the Rhinoconjunctivitis Quality of Life Questionnaire (RQLQ) score self-assessed by subjects after two and four weeks of treatment.

Safety outcomes, such as treatment-emergent adverse events (TEAEs) and treatment-related adverse events (TRAEs), associated with the excipients of azelastine hydrochloride/fluticasone propionate/sodium chloride were assessed as presented in Supplementary Material S5.

### 2.3 Statistical analysis

All statistical analyses were conducted using R software (version 4.1.2). Quantitative data were described using mean, standard deviation (SD), standard error (SE), median, minimum, and maximum. Qualitative data were described using counts and percentages. The primary outcome was analyzed using analysis of covariance (ANCOVA), with baseline scores included as covariates. The difference in mean TNSS scores between the two groups after four weeks of treatment was calculated, along with its 95% confidence interval (CI). Secondary outcomes were analyzed using two-sample *t*-tests and chi-square tests, as appropriate. These analyses were exploratory; nominal *p*-values are reported without adjustment for multiplicity, and findings are interpreted cautiously due to the increased risk of false positives. The efficacy population consisted of participants who completed the trial, had non-missing primary endpoint data, and had no major protocol violations. Missing primary endpoint values were imputed using last-observation-carried-forward (LOCF). The safety population included all participants who received at least one dose of study medication and had at least one post-baseline safety assessment; no imputation was performed for safety data, which were summarized descriptively. The exact *p*-values were reported to two significant figures, except for *p*-values below 0.001, which were reported as *p* < 0.001. A *p*-value of <0.05 indicated statistical significance.

## 3 Results

### 3.1 Patient characteristics

**Figure 1:**
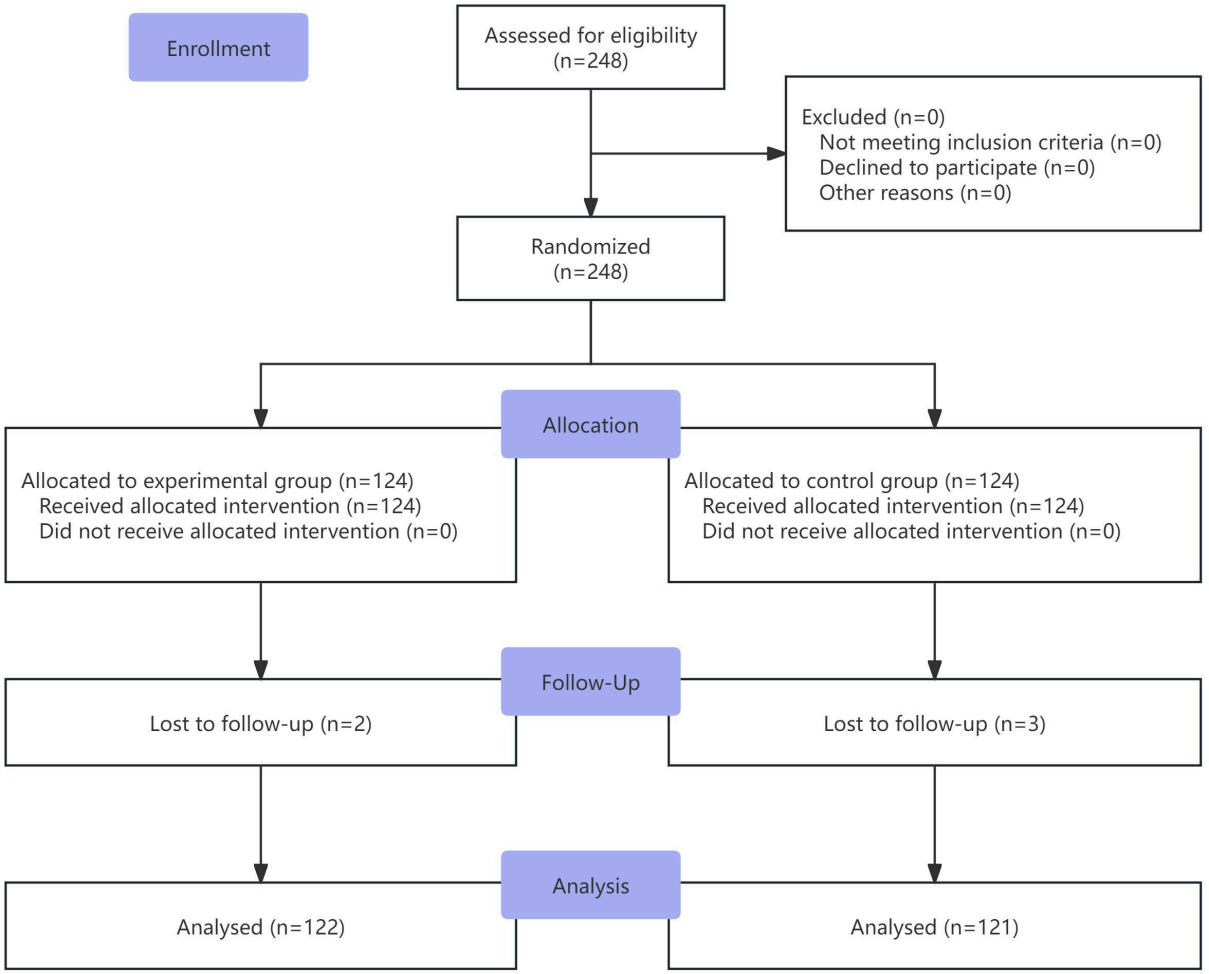
CONSORT flowchart of the study.

A total of 248 patients were enrolled and randomly assigned to the experimental group and the control group. The experimental group included 124 patients, with 2 lost to follow-up, resulting in 122 completing the trial. The control group also included 124 patients, with 3 lost to follow-up, resulting in 121 completing the trial. The flowchart of the study is shown in Figure 1. Baseline characteristics were well-balanced between the two groups (Table 1).

**Table 1:** Demographic characteristics in experimental and control groups.

Variable	Experimental group (*N* = 122)	Control group *N* = 121)	Test statistic	*p*-value
Gender			*χ*^2^ = 0.101	0.751
Male	68 (55.7%)	65 (53.7%)		
Female	54 (44.3%)	56 (46.3%)		
Age (y)			*t* = 0.587	0.558
Mean (SD)	33.6 (12.3)	32.7 (11.6)		
Range	13.0–64.0	12.0–65.0		
Height (cm)			*t* = −0.387	0.699
Mean (SD)	168.1 (8.4)	168.5 (7.7)		
Range	150.0–188.0	150.0–188.0		
Weight (kg)			*t* = 0.309	0.757
Mean (SD)	65.1 (12.4)	64.6 (12.8)		
Range	40.0–98.0	39.0–112.5		
BMI (kg/m^2^)			*t* = 0.451	0.652
Mean (SD)	22.9 (3.2)	22.7 (3.7)		
Range	16.0–31.2	15.2–37.2		
AR subtype			*χ*^2^ = 3.553	0.169
Perennial	78 (63.9%)	89 (73.6%)		
Seasonal	7 (5.7%)	8 (6.6%)		
Mixed	37 (30.3%)	24 (19.8%)		
Geographical distribution			*χ*^2^ = 0.290	0.962
Eastern China	71 (58.2%)	67 (55.4%)		
Northern China	13 (10.7%)	15 (12.4%)		
Southern China	24 (19.7%)	24 (19.8%)		
Southwestern China	14 (11.5%)	15 (12.4%)		
TNSS	10.0 ± 1.1	9.7 ± 1.3	*t* = 1.940	0.053
Rhinoscopy scores	8.6 ± 1.3	8.4 ± 1.4	*t* = 1.153	0.25
VAS	30.6 ± 3.5	29.8 ± 2.5	*t* = 2.051	0.041^a^
RQLQ	105.6 ± 16.0	101.9 ± 14.8	*t* = 1.871	0.062

a A significant difference was observed between the groups.

Eastern China: Patients were from The First Affiliated Hospital with Nanjing Medical University (*n* = 84), Tongji Hospital of Tongji University (*n* = 30), and The Second Affiliated Hospital of Zhejiang University School of Medicine (*n* = 24). Northern China: Patients were from Peking University People's Hospital (*n* = 28). Southern China: Patients were from The First Affiliated Hospital of Sun Yat-sen University (*n* = 48). Southwestern China: Patients were from Sichuan Provincial People's Hospital (*n* = 29).

AR, allergic rhinitis; SD, standard deviation.

### 3.2 Primary outcome

After four weeks of treatment, both groups showed significant reductions in TNSS scores [mean (SD)]. The scores were 2.4 (1.1) and 4.2 (1.2), respectively, compared to their baseline counterparts, which were 10.0 (1.1) and 9.7 (1.3), respectively (both *p* < 0.001). Moreover, after four weeks of treatment, the experimental group had a larger absolute value of LSmean compared to the control group. The LSmean (SE) for the control group was −5.5 (0.13), with a 95%CI of (−5.8, −5.3). For the experimental group, the LSmean (SE) was −7.6 (0.13), with a 95%CI of (−7.9, −7.4). The difference in LSmean (SE) between the two groups was −2.1 (0.19), with a 95%CI of (−2.5, −1.7). As shown in the ANCOVA analysis, the primary outcome was more obvious in the experimental group (*p* < 0.001) (Table 2).

**Table 2:** Comparative analysis of TNSS at baseline and post-treatment four weeks in experimental and control groups.

Group	Baseline mean (SD)	Outcome mean (SD)	LSmean mean (SE)	95%CI	*p*-value
Experimental group (*N* = 122)	10.0 (1.1)	2.4 (1.1)	−7.6 (0.13)	−7.9, −7.4	<0.001^a^
Control group (*N* = 121)	9.7 (1.3)	4.2 (1.2)	−5.5 (0.13)	−5.8, −5.3	<0.001^a^
Experimental group minus control group	–	–	−2.1 (0.19)	−2.5, −1.7	<0.001^b^

a Comparison of scores between baseline and after four weeks of treatment.

b Comparison after four weeks of treatment between experimental group and control group.

LSmean: The change in scores of the evaluation index after treatment compared to the corresponding baseline values.

CI, confidence interval; SD, standard deviation; SE, standard error; TNSS, total nasal symptom score.

### 3.3 Secondary outcomes

#### 3.3.1 TNSS at two weeks

After two weeks of treatment, the LSmean (95%CI) was −6.0 (−6.3, −5.8) in the experimental and −3.5 (−3.7, −3.2) in the control group, with a difference of −2.6 (−2.9, −2.2), indicating that outcome was better in the experimental group (*p* < 0.001). Moreover, the absolute difference in LSmean after two weeks was larger than that after four weeks of treatment, suggesting that the therapeutic effect appeared earlier in the experimental group than in the control group (Table 3).

**Table 3:** Comparative analysis of TNSS at baseline and post-treatment two weeks in experimental and control groups.

Group	Baseline mean (SD)	Outcome mean (SD)	LSmean mean (SE)	95%CI	*p*-value
Experimental group (*N* = 122)	10.0 (1.1)	4.0 (1.2)	−6.0 (0.12)	−6.3, −5.8	<0.001^a^
Control group (*N* = 121)	9.7 (1.3)	6.2 (1.4)	−3.5 (0.12)	−3.7, −3.2	<0.001^a^
Experimental group minus control group	–	–	−2.6 (0.17)	−2.9, −2.2	<0.001^b^

a Comparison of scores between baseline and after two weeks of treatment.

b Comparison after two weeks of treatment between experimental group and control group.

LSmean: The change in scores of the evaluation index after treatment compared to the corresponding baseline values.

CI, confidence interval; SD, standard deviation; SE, standard error; TNSS, total nasal symptom score.

#### 3.3.2 Individual item scores in TNSS at two and four weeks

**Figure 2:**
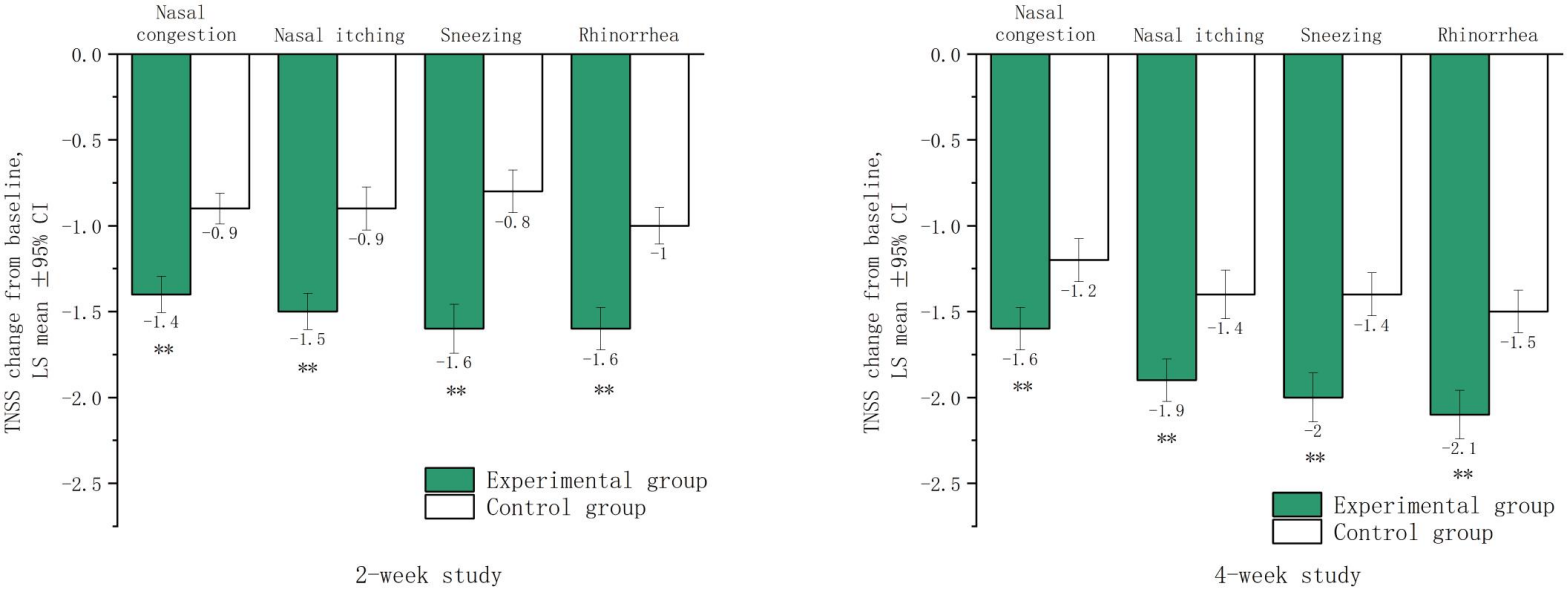
Lsmean of subscores in the TNSS after two and four weeks of treatment. LSmean: The change in subscores of TNSS after treatment compared to baseline values. **The difference between the experimental group and the control group was statistically significant (*p* < 0.001).

After two and four weeks of treatment, the LSmean (SD) values for nasal congestion were −1.4 (0.6) and −1.6 (0.7) in the experimental group, −0.9 (0.5) and −1.2 (0.7) in the control group, respectively. For nasal itching, the LSmean (SD) values were −1.5 (0.6) and −1.9 (0.7) in the experimental group, −0.9 (0.7) and −1.4 (0.8) in the control group, respectively. For sneezing, the LSmean (SD) values were −1.6 (0.8) and −2.0 (0.8) in the experimental group, compared to −0.8 (0.7) and −1.4 (0.7) in the control group, respectively. For rhinorrhea, the LSmean (SD) values were −1.6 (0.7) and −2.1 (0.8) in the experimental group, −1.0 (0.6) and −1.5 (0.7) in the control group, respectively. These data suggested that significant therapeutic effects were achieved in both groups after two and four weeks of treatment, and more significant in the experimental group (Figure 2).

#### 3.3.3 Rhinoscopy scores at four weeks

**Figure 3:**
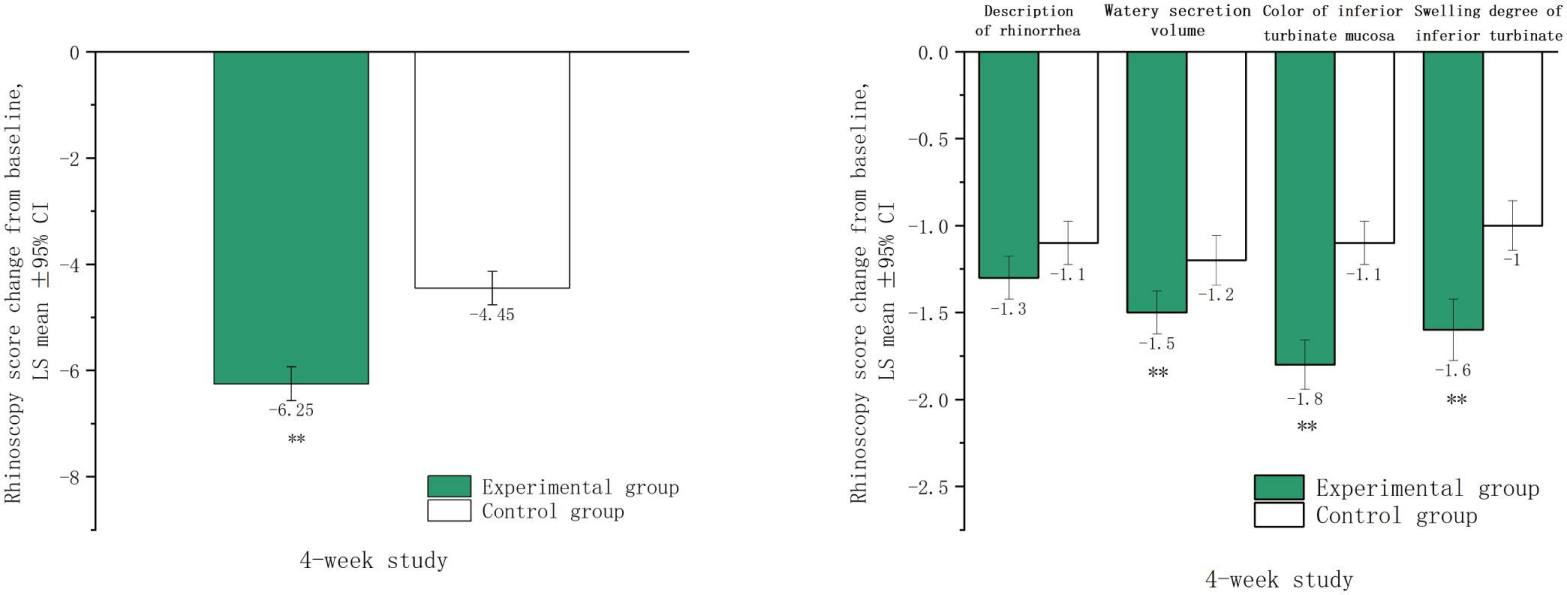
Rhinoscopy scores after four weeks of treatment. LSmean: The change in rhinoscopy scores after treatment compared to baseline values. **The difference between the experimental group and control group the was statistically significant (*p* < 0.001).

After four weeks of treatment, significant reductions in total rhinoscopy scores were observed in both groups compared to baseline (*p* < 0.001). The experimental group showed a greater improvement, with an LSmean (SD) score reduction from 8.6 (1.3) to 2.4 (1.7), compared to the control group, which reduced from 8.4 (1.4) to 4.0 (1.6). The difference in LSmean (95%CI) between the groups was −1.8 (−2.2, −1.3), indicating more significant alleviation in the experimental group (*p* < 0.001). The experimental group also exhibited greater improvements in inferior turbinate swelling, mucosal color, and watery secretion volume (all *p* < 0.001), but no significant difference was observed in description of rhinorrhea (*p* = 0.088) (Figure 3).

#### 3.3.4 Trends in daily VAS total scores during two weeks

**Figure 4:**
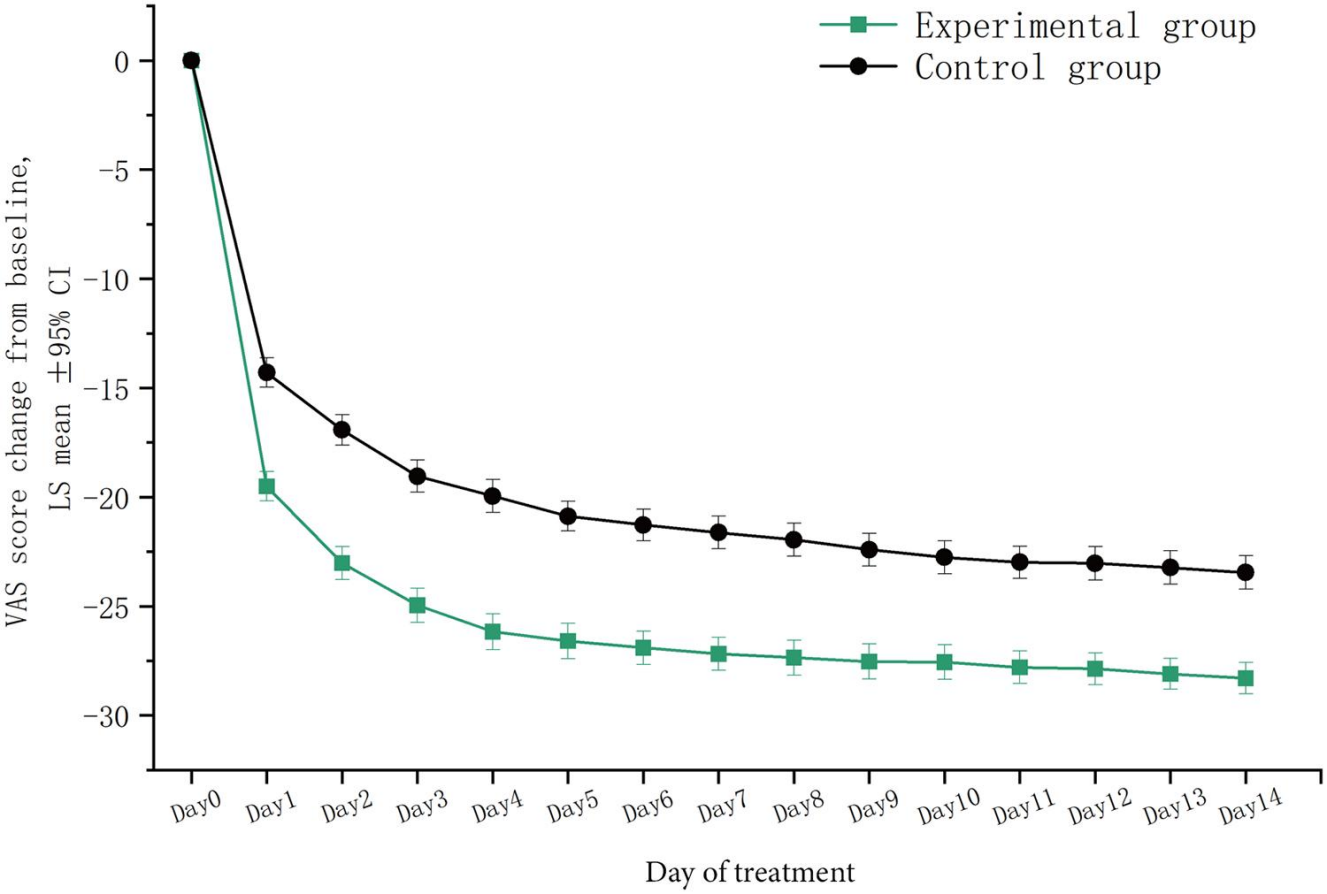
Trends in VAS total scores within two weeks of treatment. LSmean, the change in VAS total scores after treatment compared to baseline values; VAS, visual analogue scale.

After two weeks of treatment, the VAS total score [mean (SD)] decreased significantly in either the experimental group [2.3 (1.6) vs. 30.6 (3.5)] or the control group [6.5 (3.5) vs. 30.0 (2.3)] compared to their baseline values (all *p* *<* 0.001). During this treatment period, the daily VAS total score showed a downward trend in both groups. Compared to the baseline, the score decreased significantly on the first day of treatment in the experimental group (*p* *<* 0.001), and this decrease was larger than that in the control group (*p* *<* 0.001). This therapeutic advantage maintained during the two weeks. In addition, the decrease in each individual VAS score was also greater in the experimental group (All *p* *<* 0.001) (Figure 4).

#### 3.3.5 Changes in RQLQ scores during two and four weeks

Compared to baseline, total RQLQ scores [mean (SD)] and all subscores significantly decreased in both groups after two and four weeks of treatment (*p* < 0.001). At two weeks, the experimental group showed a greater reduction in total RQLQ score [−80.0 (18.5)] than the control group [−50.0 (15.4)], with the largest decreases observed in non-nasal/ocular symptom and nasal symptom scores. At four weeks, the experimental group continued to show a more significant reduction in total RQLQ score [−88.9 (17.5)] compared to the control group [−72.7 (20.0)] (*p* < 0.001).

**Figure 5:**
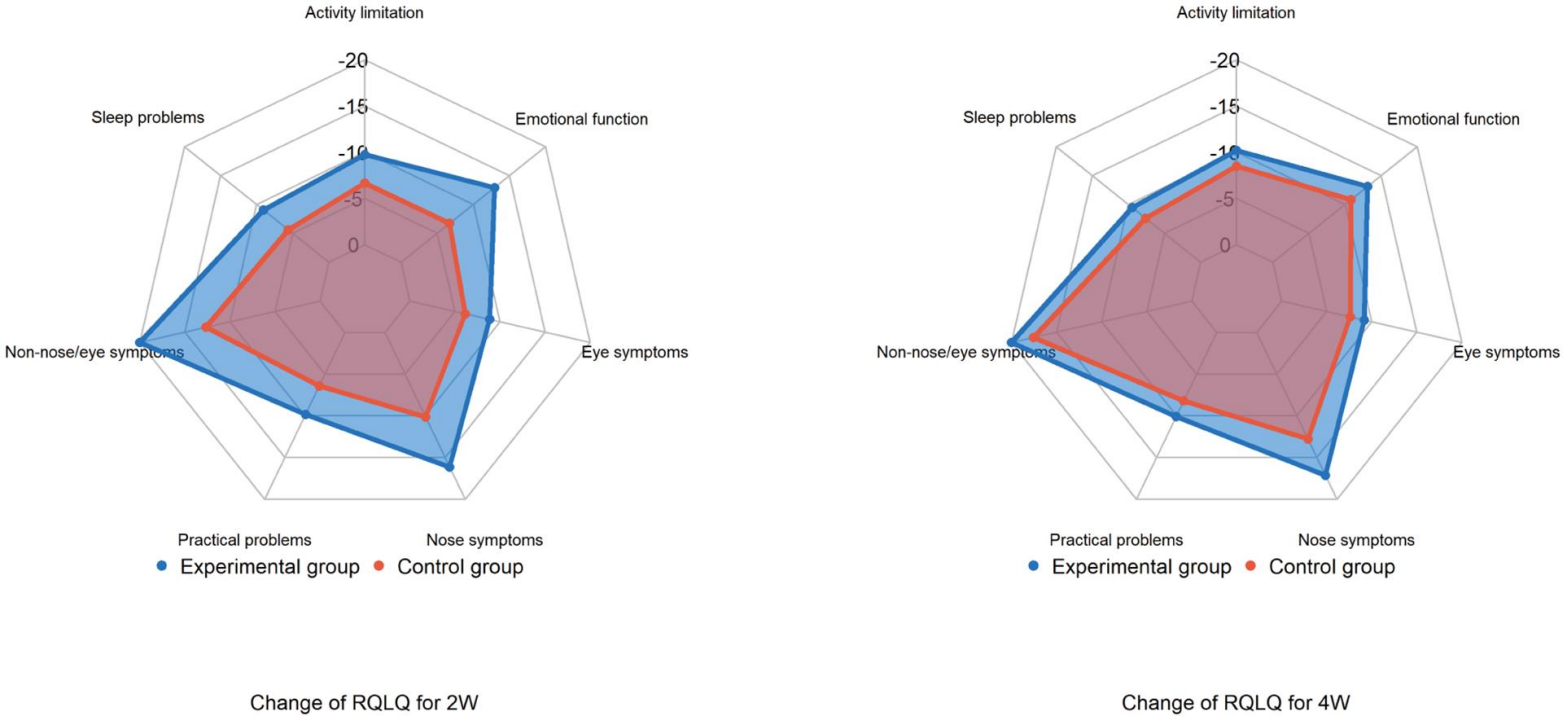
Changes in RQLQ total scores and subscores after treatment. RQLQ, rhinoconjunctivitis quality of life questionnaire.

The RQLQ includes seven subscores: activity limitation, sleep problems, nose symptoms, eye symptoms, non-nasal/eye symptoms, practical problems, and emotional function. At both two and four weeks post-treatment, all subscores decreased in both groups, with non-nasal/ocular symptoms showing the largest reductions. The experimental group exhibited more significant improvements across all subscores at two weeks compared to the control group (Figure 5).

### 3.4 Exploratory clinical outcomes

The patients were stratified into three subgroups according to AR subtypes. After four weeks of treatment, the LSmean (SD) values in TNSS total scores were −7.6 (1.4) in the experimental subgroup and −5.6 (1.5) in the control subgroup of perennial AR; −7.6 (1.4) in the experimental subgroup and −5.1 (1.1) in the control subgroup of seasonal AR; and −7.6 (1.7) in the experimental subgroup and −5.2 (1.3) in the control subgroup of mixed AR. After four weeks of treatment, the TNSS total scores dropped more evidently in all experimental subgroups stratified by AR subtypes (all *p* < 0.001) (Table 4).

**Table 4:** TNSS variations from baseline to post-treatment four weeks across three AR subgroups.

Group	Index	Perennial AR	Seasonal AR	Mixed AR	Total
Experimental subgroup	*N*	80	7	37	124
	Mean (SD)	−7.6 (1.4)	−7.6 (1.4)	−7.6 (1.7)	−7.6 (1.5)
	Range	−11.0 to −3.0	−9.0 to −5.0	−12.0 to −4.0	−12.0 to −3.0
	Missing	2 (2.50%)	0	0	2 (1.61%)
Control subgroup	*N*	92	8	24	124
	Mean (SD)	−5.6 (1.5)	−5.1 (1.1)	−5.2 (1.3)	−5.5 (1.4)
	Range	−9.0 to −2.0	−7.0 to −3.0	−8.0 to −2.0	−9.0 to −2.0
	Missing	3 (3.26%)	0	0	3 (2.42%)

AR, allergic rhinitis; SD, standard deviation; TNSS, total nasal symptom score.

**Figure 6:**
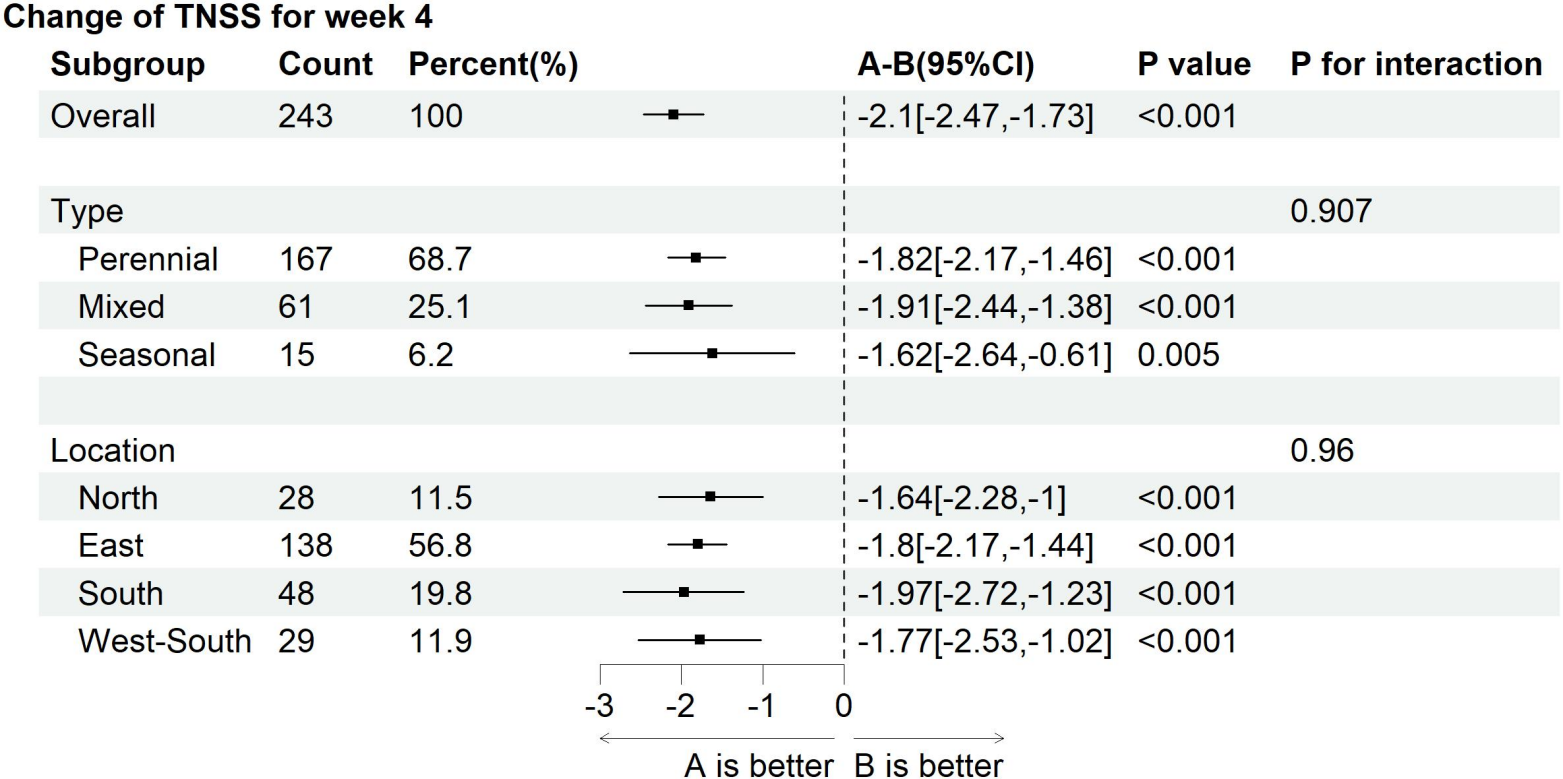
Subgroup analysis between the experimental group **(A)** and the control group **(B)**.

The forest plot based on subgroup analysis adjusted for baseline showed no interaction effects between AR phenotype and geographical region on the change in TNSS total scores from baseline to post-treatment four weeks (Figure 6).

### 3.5 Adverse events

Throughout the entire trial period, 15 patients in the experimental group experienced TEAEs, with an incidence of 12.1%, and 10 experienced TRAEs, with an incidence of 8.1%. In the control group, 20 patients experienced TEAEs, with an incidence of 16.1%, and 13 experienced TRAEs, with an incidence of 10.5%. The chi-square test showed no significant difference in the incidence of adverse reactions between the two groups (TEAE: *P* = 0.347; TRAE: *p* = 0.498). Among these events, common cold was the most frequent, with an incidence of 4.0% in the experimental group and 4.8% in the control group. The incidence of TRAEs in the experimental group was equal to or lower than that in the control group, except for taste disturbances (bitter taste). All TRAEs were mild, and patients could recover without intervention. During the entire study period, neither serious adverse events related to trial medications, nor withdrawals due to drug-related adverse events were reported (Table 5).

**Table 5:** Incidence of TEAEs and TRAEs in the experimental and control groups.

TEAEs			TRAEs		
Events	Experimental group (*N* = 124)	Control group (*N* = 124)	Events	Experimental group (*N* = 124)	Control group (*N* = 124)
Common cold	5 (4.0%)	6 (4.8%)	Taste disorders (bitter taste)	3 (2.4%)	2 (1.6%)
Taste disorders (bitter taste)	3 (2.4%)	2 (1.6%)	Epistaxis	3 (2.4%)	4 (3.2%)
Epistaxis	3 (2.4%)	4 (3.2%)	Pharyngeal irritation	2 (1.6%)	3 (2.4%)
Fever	2 (1.6%)	2 (1.6%)	Headache	2 (1.6%)	2 (1.6%)
Pharyngeal irritation	2 (1.6%)	3 (2.4%)	Nasal itching	2 (1.6%)	2 (1.6%)
Headache	2 (1.6%)	2 (1.6%)	Cough	1 (0.8%)	2 (1.6%)
Nasal itching	2 (1.6%)	2 (1.6%)	Dizzy	0 (0.0%)	1 (0.8%)
Xerostomia	1 (0.8%)	0 (0.0%)	Nausea	0 (0.0%)	1 (0.8%)
Cough	1 (0.8%)	2 (1.6%)			
Drowsiness	1 (0.8%)	2 (1.6%)			
Eye itching	1 (0.8%)	0 (0.0%)			
Diarrhea	1 (0.8%)	0 (0.0%)			
Dizzy	0 (0.0%)	1 (0.8%)			
Nausea	0 (0.0%)	1 (0.8%)			
Snore	0 (0.0%)	1 (0.8%)			
Rash	0 (0.0%)	1 (0.8%)			
Tinnitus	0 (0.0%)	1 (0.8%)			

TEAEs, treatment-emergent adverse events; TRAEs, treatment-related adverse events.

Multiple TEAEs and TRAEs might occur simultaneously in one case.

## 4 Discussion

This multicenter, randomized, controlled trial evaluated the efficacy and safety of combining nasal saline irrigation with Aze-Flu nasal spray in managing moderate-to-severe PAR. After four weeks of treatment, the experimental group showed a significant reduction in TNSS compared to the control group. The combination therapy also led to more significant improvements in nasal signs, quality of life, and a comparable safety profile. These findings highlight the clinical benefits of integrating nasal saline irrigation into standard treatment regimens for PAR, particularly in primary care settings where cost-effective and accessible interventions are highly valued.

However, several limitations should be acknowledged. Firstly, the study population was limited to patients aged 12 years and above, restricting the applicability of the findings to younger children. Secondly, the lack of a placebo control group means that the placebo effect might have influenced the observed outcomes. Additionally, the four-week study duration is relatively short, and longer-term follow-up is needed to evaluate the sustained efficacy and safety of the treatment regimen. The open-label design carries inherent performance-bias risk; although rhinoscopic evaluations were performed by blinded assessors, patient-reported outcomes (TNSS, VAS, and RQLQ) remain susceptible to expectation bias. Finally, drug-delivery confounding is a potential limitation: the experimental group used a fixed-dose combination (FDC) that may improve nasal deposition, pharmacodynamic synergy, and adherence compared with two separate sprays. These advantages could partly account for the observed improvements independent of saline irrigation. Future three-arm studies (FDC + irrigation, FDC alone, separate sprays + irrigation) are needed to isolate the specific contribution of saline irrigation.

Nasal saline irrigation is a safe, simple, and practical adjuvant treatment method, believed to have effects such as diluting mucus, improving mucociliary clearance, reducing mucosal edema, and decreasing allergen load in the nasal and sinus cavities (16, 18, 24). It is widely used in the management of AR, chronic rhinosinusitis, and post-nasal and sinus surgery (25–27). Our findings align with and advance the existing literature on AR management. Previous studies have shown nasal saline irrigation can improve symptoms of AR (20–23), but evidence for its combined use with pharmacological treatments has been limited and often of low quality. The Cochrane meta-analysis, which reviewed 14 studies involving 747 subjects, suggested that nasal saline irrigation may reduce patient-reported disease severity for up to three months, but the quality of evidence was assessed as low or very low (17). Our study addresses these limitations by employing a large, multicenter, randomized controlled trial design, providing high-quality evidence for the efficacy and safety of the combination therapy.

The two-stage nasal saline irrigation protocol used in this study is innovative. The initial use of hypertonic saline for rapid symptom relief, followed by isotonic saline to minimize side effects, represents a balanced approach that optimizes patient outcomes. This strategy is supported by evidence that hypertonic saline provides quicker symptom relief while isotonic saline is better tolerated over longer periods (28). Our study demonstrates that this protocol, when combined with Aze-Flu nasal spray, results in significant improvements in symptom scores and quality of life measures, surpassing the benefits of pharmacological treatments alone.

The study's strengths include its multicenter design, involving six clinical centers across different regions in China, ensuring a diverse and representative patient sample. The large sample size of 248 patients provides sufficient statistical power to detect significant differences between the treatment groups. Additionally, the low dropout rate of 2.02% (5 out of 248 cases) indicates high patient compliance and excellent data integrity, further bolstering the credibility of the study outcomes.

The findings have important implications for clinical practice and healthcare policy. In primary care settings, nasal saline irrigation offers a valuable adjunct to pharmacological treatments. The combination therapy demonstrated in this study provides significant symptom relief and quality of life improvements without increasing adverse events, making it a practical and effective option for managing moderate-to-severe PAR. Clinicians can confidently integrate nasal saline irrigation into their treatment protocols, knowing that it enhances the efficacy of existing treatments and is well-tolerated by patients.

## 5 Conclusions

This study provides compelling evidence for the efficacy and safety of combining nasal saline irrigation with Aze-Flu nasal spray in managing moderate-to-severe PAR. The findings highlight the importance of integrating this cost-effective and accessible intervention into primary care settings, offering significant benefits for patients suffering from this debilitating condition. Future research should build on these findings to further refine treatment protocols and explore the potential of this combination therapy in different patient populations.

## Data Availability

The datasets presented in this article are not readily available because. The datasets generated and analysed during the current study are not publicly available due to privacy or ethical restrictions but may be available from the corresponding author on reasonable request. Requests to access the datasets should be directed to Lei Cheng, chenglei@jsph.org.cn.
