# Supplementary material for: Nasal saline irrigation with azelastine-fluticasone nasal spray in moderate-to-severe persistent allergic rhinitis: a randomized controlled trial
Source: Front Allergy. 2025 Oct 13;6:1622510. doi: 10.3389/falgy.2025.1622510 (PMC12554748; doi:10.3389/falgy.2025.1622510)
Supplement: Supplementary file 1 [file Datasheet1.docx]

**Supplementary Material 1**

**Inclusion, exclusion, termination, and withdrawal criteria**

**Inclusion criteria**

1. Aged 12 years and above.
2. Meeting the diagnostic criteria for allergic rhinitis (AR) as outlined in the "Chinese Guidelines for Diagnosis and Treatment of Allergic Rhinitis (2022, Revised Edition)":
3. Symptoms: Presence of two or more of the following: sneezing, watery rhinorrhea, nasal itching, and nasal congestion. These symptoms should last or accumulate to more than 1 hour per day and may be accompanied by ocular symptoms such as itching, tearing, and redness.
4. Signs: Pale and edematous nasal mucosa, and watery nasal discharge.
5. Allergen tests: Prior to signing the informed consent, patients should have a positive skin prick test (SPT) or serum specific IgE for at least one allergen within the previous 12 months. a) If the patient exhibits a hypersensitivity to a particular allergen, a wheal or flare will occur at the puncture site within 20 minutes, with an average diameter greater than 3 mm compared to the negative control, which is considered a positive SPT. The intensity of SPT reaction can be assessed using the skin index (SI), including the longest diameter and the longest perpendicular diameter of the allergen and histamine wheals, avoiding pseudopods, and the average diameter of the wheal. The ratio of two average diameters is calculated as SI in four grades: + for 0.3 ≤ SI < 0.5; ++ for 0.5 ≤ SI < 1.0; +++ for 1.0 ≤ SI < 2.0; ++++ for SI ≥ 2.0. b) A threshold of serum IgE level is set at 0.35 kU_A_/L, with a value equal or greater considered positive, indicating a sensitized state of the body. The results of serum specific IgE testing are divided into seven grades, with less than 0.35 kU_A_/L as grade 0; 0.35 to 0.69 kU_A_/L as grade 1; 0.7 to 3.4 kU_A_/L as grade 2; 3.5 to 17.4 kU_A_/L as grade 3; 17.5 to 49.9 kU_A_/L as grade 4; 50 to 100 kU_A_/L as grade 5; greater than 100 kU_A_/L as grade 6. Patients with an SPT at ++ or higher, or serum specific IgE levels in grade 1 or higher are eligible for this study.
6. Moderate to severe AR, with a total nasal symptom score of ≥ 6 points at the first follow-up visit, significantly affecting the quality of daily life and defined as having one or more of the following symptoms:
7. Sleep disturbances.
8. Limitations in daily activities, leisure, and/or exercise.
9. Impediments to learning or work.
10. Distress symptoms.
11. A history of allergic rhinitis/rhinoconjunctivitis (seasonal and/or perennial) for at least 1 year before enrollment.
12. Previous treatment with monotherapy with antihistamines or corticosteroids, but with symptoms not fully controlled.
13. No concomitant diseases or treatments that may interfere with the interpretation of study results.
14. Appropriate medical contraceptive measures for women of childbearing age during the study period and for 4 weeks after the end of the trial.
15. Good compliance, voluntary participation in this clinical study, and signature on an informed consent form.

**Exclusion criteria**

1. Presence of nasal mucosal erosion, nasal ulcers, or nasal septal perforation.
2. Nasal diseases that may affect drug deposition or assessment, such as sinus infections, nasal polyps, or severe nasal septal deviation.
3. History of nasal or sinus surgery within the past 12 months.
4. Ongoing immunotherapy (a 6-month clearance period required after the last dose of immunotherapy).
5. Local oropharyngeal candidiasis within 30 days prior to screening.
6. Glaucoma or cataracts.
7. Active or quiescent pulmonary tuberculosis, as well as untreated local or systemic fungal, bacterial, viral, parasitic, or herpes simplex infections.
8. Severe pulmonary diseases, such as COPD.
9. Moderate to severe asthma, or mild persistent asthma (patients with intermittent asthmatic conditions may be included).
10. Arrhythmias or heart diseases requiring treatment.
11. Active hepatitis B or C, or with clinically significant results in tests for antibodies to human immunodeficiency virus or Treponema pallidum.
12. Any condition requiring the use of sedative drugs or centrally acting "contraindicated medications" (such as tricyclic antidepressants).
13. Use of investigational drug (including azelastine hydrochloride or fluticasone propionate) within 30 days prior to screening.
14. Chronic sinus infections (more than three episodes per year).
15. Allergies to the study medication or its excipients.
16. Severe sodium chloride metabolism disorders and allergies.
17. Preparation for pregnancy or breastfeeding for women.
18. Upper respiratory tract infections within 2 weeks prior to the first visit, such as the common cold, influenza, or sinus infections.
19. Current participation in another clinical trial, or any other drug clinical trial within 3 months prior to screening.
20. Current use or anticipated need for ritonavir or potent cytochrome P450 3A4 inhibitors, including medications containing cobicistat.
21. Presence of alcohol abuse or drug misuse that may affect the implementation of the study.
22. Any other condition deemed by the investigator as unsuitable for the subject to participate in the trial.
23. Failure to participate in the washout period as per the protocol.

**Termination criteria**

If a subject meets any of the following criteria, medication is discontinued, and safety follow-up is completed:

1. Clinical adverse events, laboratory test abnormalities, or other medical conditions that the investigator considers to discontinue the medication in the best interest of the subject.
2. Pregnancy.
3. Any reason failing the enrolled patient to complete the study.
4. Poor medication compliance.
5. Participation in another clinical trial (defined as having signed the informed consent to another trial).
6. After enrollment in the study, the patient requests to withdraw for any reason.

**Withdrawal criteria**

1. The subject refuses to continue treatment and/or observation, and withdraws informed consent during the trial.
2. The investigator allows a subject to withdraw from the study due to adverse events or other reasons.
3. The investigator deems that withdrawal from the trial is in the best interest of the subject.
4. Situations where withdrawal due to adverse events is permitted as stipulated by the protocol.

**Supplementary Material 2**

**Screening period, washout period, and experimental procedure chart**

**Screening period (Days -7 to -4)**

During the screening period, the following tasks are completed:

1. Signing of the Informed Consent Form.
2. Collection of demographic data (gender, age, height, weight, etc.).
3. Collection of medical and treatment history (such as records of rhinitis, drug allergy, use of corticosteroids and antihistamines, and immunotherapy, etc., during at least one year earlier).
4. Vital signs examination: Temperature, respiration, heart rate, blood pressure.
5. Physical examination (including skin and mucous membranes, lymph nodes, head, neck, chest, abdomen, spine/extremities, oropharynx, eyes).
6. Specialized examination (allergen SPT/specific IgE).
7. Virological tests: Hepatitis B panel, hepatitis C virus antibody, antibody to human immunodeficiency virus or antibody to Treponema pallidum-specific antibody.
8. Laboratory tests: Acceptable results from tests conducted within 7 days prior to screening, no need for repeating testing:
9. Complete blood count: White blood cell (WBC) count, red blood cell (RBC) count, hemoglobin (Hb), platelet (PLT) count.
10. Urinalysis: Qualitative or quantitative leukocytes, qualitative or quantitative erythrocytes, pH, qualitative protein (PRO).
11. Blood biochemistry: Alanine aminotransferase (ALT), aspartate aminotransferase (AST), total bilirubin (TBIL), urea, creatinine (Cr).
12. Electrocardiogram: 12-lead electrocardiogram.
13. Urine pregnancy test: For women of reproductive age (15-49 years, excluding postmenopausal or surgically sterilized women).
14. Records of concomitant medications.

**Washout period (Days -3 to -1)**

The washout period refers to the period during which subjects discontinue the use of medications not permitted in the study prior to trial commencement, including first-line and second-line drugs recommended by guidelines for AR. The first-line treatment drugs encompass intranasal corticosteroids, second-generation oral and intranasal antihistamines, and oral leukotriene receptor antagonists. The second-line treatment drugs include oral corticosteroids, oral and intranasal mast cell stabilizers, intranasal decongestants, and intranasal anticholinergic drugs; as well as traditional Chinese medicines containing known active ingredients (such as antihistamines, ephedrine, or pseudoephedrine) and IgE antagonists.

The purpose of setting a washout period period in this study is to allow the body to clear previous drugs that may affect study outcomes. The length of the washout period should be determined based on disease type, drug half-lives, and safety.

If a subject is under no medication currently, a washout period is not required, and the screening period starts from Day -7 to Day -1.

**Experimental procedure chart**

| Time  Item | Screening period | Washout period | Treatment period  (4 weeks) | | Post-treatment | | Early Withdrawal |
| --- | --- | --- | --- | --- | --- | --- | --- |
|  |  |  | Follow-up 1 | Follow-up 2 | Follow-up 3 | Phone Follow-up |  |
|  | Days  -7 to -4 | Days  -3 to -1 | Day 1 | Days  15 ± 2 | Days  29 ± 2 | Post-treatment week +1 ± 2 days |  |
| Informed consent | **√** |  |  |  |  |  |  |
| Demographics | **√** |  |  |  |  |  |  |
| Medical and treatment history | **√** |  |  |  |  |  |  |
| Vital signs | **√** |  |  |  | **√** |  | **√** |
| Physical examination | **√** |  |  |  | **√** |  | **√** |
| Allergy test | **√** |  |  |  |  |  |  |
| Virological test | **√** |  |  |  |  |  |  |
| Laboratory test | **√** |  |  |  | **√** |  | **√** |
| Medication as per group |  |  | **√** | **√** |  |  |  |
| Concomitant medication | **√** |  | **√** | **√** |  |  |  |
| Rhinoscopy |  |  | **√** |  | **√** |  | **√** |
| TNSS |  |  | **√** | **√** | **√** |  | **√** |
| VAS |  |  | **√** | **√** |  |  | **√** |
| RQLQ |  |  | **√** | **√** | **√** |  | **√** |
| Adverse events |  |  | **√** | **√** | **√** | **√** | **√** |
| Medication dispensing and diary collection |  |  | **√** | **√** | **√** |  | **√** |

- TNSS, total nasal symptom score; RQLQ, rhinoconjunctivitis quality of life questionnaire; VAS, visual analogue scale.
- During the treatment period, subjects are followed up at the outpatient department on the first day of treatment (Follow-up 1) and again during the second week to assess medication use, evaluate efficacy, and record adverse events.
- Post-treatment follow-up involves an outpatient visit after the end of treatment and a telephone follow-up one week later to evaluate treatment efficacy and gather information on adverse events experienced by the subjects.
- Allergy test: Allergy reactions are assessed using either SPT or serum specific IgE assay. Specific IgE test results within the last 12 months prior to signing the informed consent are accepted. However, SPT results are only accepted if they are from the same center and within the last 12 months prior to informed consent.
- Screening period: Complete blood count, urinalysis, blood biochemistry, and electrocardiogram are performed, and a pregnancy test is also required for women of a reproductive age. Results from tests conducted within 7 days prior to signing the informed consent are acceptable, and no repeat tests are needed. These tests should be repeated at Follow-up 3 after the end of treatment.
- Virological test: Results from tests conducted within 4 weeks prior to signing the informed consent are acceptable.
- If a subject is not currently taking any medication (medication not allowed in the trial), a washout period is not set, and the screening period starts from Day -7 to Day -1.
- If a subject cannot complete the 4-week treatment and withdraws from the trial, a safety and efficacy should be evaluated.

**Preparation of the hypertonic and isotonic saline**

Hypertonic and isotonic saline solutions are typically composed of a dropper bottle and an irrigation solution. The dropper bottle is manufactured from polyethylene material, while the irrigation solution consists of sea salt and purified water. For hypertonic seawater nasal irrigation solution, the sodium chloride (NaCl) concentration is 2.3%, with a pH range of 5.5–7.0, sodium chloride content of 22–24 g/L, heavy metal content (expressed as Pb) < 10 μg/mL, and total bacterial colony count < 20 CFU/mL. For physiological seawater nasal irrigation solution, the sodium chloride concentration is 0.9%, with a pH range of 5.5–7.0, sodium chloride content of 8.5–9.5 g/L, heavy metal content (expressed as Pb) < 10 μg/L, and total bacterial colony count < 20 CFU/mL. This product is classified as a Class II medical device and is manufactured using Blow-Fill-Seal (BFS) aseptic production technology. Its inverted design prevents leakage and minimizes the risk of bacterial contamination.

**Supplementary Material 3**

**Follow-up methods and withdrawal mechanism**

**During-treatment follow-up tasks (Days 1 to 28)**

1. On the first day of treatment (follow-up 1), the subject visits the hospital for examination and is provided with therapeutic drugs and a diary card.
2. At the end of the second week of treatment (follow-up 2), an outpatient follow-up is conducted to assess the subject's medication use and adverse events.
3. The TNSS is used to assess nasal symptoms, including nasal congestion, itching, rhinorrhea, and sneezing. The score is recorded by the investigator on the first day of treatment (follow-up 1) and at the end of the second week (follow-up 2).
4. The VAS is used to assess nasal symptoms, including nasal congestion, itching, rhinorrhea, and sneezing. The subject records the score daily during the first two weeks of treatment, starting from the first day (follow-up 1).
5. The RQLQ assesses items including daily activities, sleep, non-nasal-eye symptoms, rhinitis-related behaviors, nasal symptoms, ocular symptoms, and emotional responses. The subject self-assesses once on the first day of treatment (follow-up 1) and at the end of the second week (follow-up 2).
6. On the first day of treatment (follow-up 1), the investigator performs rhinoscopy to assess the nasal condition of the subject.

**Post-treatment follow-up tasks (Day 29 ± 2)**

1. Schedule the subject’s visit to the clinic, document their medication use, assess treatment efficacy and adverse events, and collect diary cards.
2. Vital signs examination: Body temperature, respiratory rate, heart rate, blood pressure.
3. Physical examination (including skin, mucous membranes, lymph nodes, head, neck, chest, abdomen, spine/extremities, oropharynx, eyes).
4. Calculate the TNSS.
5. Complete the RQLQ.
6. Complete the rhinoscopy examination.
7. Laboratory tests:
8. Routine blood indexes: White blood cell count, red blood cell count, hemoglobin, platelet count.
9. Urinalysis: Qualitative or quantitative detection of leukocytes, qualitative or quantitative detection of erythrocytes, pH level, qualitative detection of protein.
10. Blood biochemistry: Alanine aminotransferase, aspartate aminotransferase, total bilirubin, urea, creatinine.
11. Electrocardiogram: 12-lead ECG.
12. Urine pregnancy test: For women of reproductive age (15-49 years old, excluding postmenopausal or surgically sterilized women).

**Post-treatment telephone follow-up**

On Day 35 ± 2 after the end of treatment, a telephone follow-up is conducted to inquire about adverse events experienced by the subjects.

**Unscheduled follow-up**

During the screening period (Days -7 to -4), washout period period (Days -3 to -1), and treatment period (Days 1 to 28), follow-ups are scheduled on Day 1, Day 15 ± 2, and Day 29 ± 2, with a telephone follow-up at 1 week ± 2 days after the end of treatment. Unscheduled follow-ups may be conducted if necessary.

**Concomitant medications**

Subjects will not be allowed to receive the following medications concurrently throughout the study. All information regarding concomitant medications used during the study (generic names of drugs, purpose of administration, dosage, and timing) is documented in the Case Report Form (CRF).

1. Antihistamines (including oral formulations, nasal preparations, and ophthalmic preparations, as well as sleep and appetite aids and cold remedies).
2. Corticosteroids.
3. Hypertonic/physiological saline preparations.
4. Oral and intranasal anticholinergic medications.
5. Leukotriene receptor antagonists
6. Oral antibiotics.
7. Mast cell stabilizers.
8. Medications containing ephedrine or pseudoephedrine.
9. Decongestants (including cold remedies).
10. Traditional Chinese medicines containing known active ingredients (such as antihistamines, ephedrine, or pseudoephedrine).
11. IgE antagonists.
12. Immunosuppressants/immunomodulators.

**Supplementary Material 4**

**Rhinoscopy scoring methods**

| Item | None  (score 0) | Mild  (score 1) | Moderate  (score 2) | Severe  (score 3) |
| --- | --- | --- | --- | --- |
| Degree of inferior turbinate swelling | None | Visible center of middle turbinate | Between score 1 and 3 | Invisibility of the middle turbinate |
| Color of inferior turbinate mucosa | Normal | Light red | Red | Pale |
| Watery secretion volume | None | Only adhesion | Between score 1 and 3 | Fullness |
| Description of rhinorrhea | None | Aqueous | Viscous | Purulent |

Rhinoscopy score is the sum of four individual item scores. Thus, the maximum total score for the severity of symptoms observed through anterior rhinoscopy is 12. A decrease in symptom severity score is represented by a negative value. A larger absolute of this negative value indicates a better outcome.

Outcome assessors were blinded as follows:

- A dedicated clinical research coordinator (CRC) escorted each patient throughout every follow-up visit.
- Patients first completed the TNSS, RQLQ and VAS in a separate room with the CRC; the questionnaires were collected before any clinical encounter.
- The physician who performed rhinoscopy was not informed of the patient’s group assignment and had no access to the questionnaires at the time of examination.
- Rhinoscopy scores were recorded on a standardized form that contained only the patient ID, ensuring assessor blinding.

**Supplementary Material 5**

**Assessment method for adverse events to azelastine hydrochloride/fluticasone propionate/sodium chloride/loratadine and related pharmaceutical excipients**

**Adverse event assessment**

Adverse events associated with azelastine hydrochloride, fluticasone propionate, sodium chloride, loratadine, and related pharmaceutical excipients are evaluated. Given that these events are spontaneously reported by users of varying sizes, it may not be possible to accurately assess the frequency of these adverse events or to determine the causal relationship between drug exposure and the adverse events.

1. Azelastine fluticasone nasal spray

Adverse events include:

1. Cardiac disorders: Atrial fibrillation, tachycardia, palpitations.
2. Eye disorders: Blurred vision, cataracts, conjunctivitis, dry eyes and eye irritation, eye swelling, glaucoma, increased intraocular pressure, visual abnormalities, dry eye syndrome.
3. Gastrointestinal system disorders: Nausea, vomiting.
4. General disorders and administration site conditions: Pain, application site irritation, chest pain, facial and tongue edema, fatigue, intolerance.
5. Immune system disorders: Allergic reactions/anaphylactoid reactions, which in very rare cases may be severe, hypersensitivity reactions.
6. Musculoskeletal system disorders: Growth inhibition.
7. Nervous system disorders: Disturbances or loss of smell and/or taste, dizziness, involuntary muscle contractions, paresthesia, dysosmia.
8. Psychiatric disorders: Anxiety, confusion, tension.
9. Urinary system disorders: Urinary retention.
10. Respiratory system disorders: Bronchospasm, cough, dysphonia, dyspnea, hoarseness, nasal septum perforation, nasal discomfort, nasal dryness, nasal ulcers, nasal sores, throat pain, throat dryness and throat irritation, voice changes, wheezing.
11. Skin disorders: Angioedema, erythema, facial swelling, pruritus, rash, urticaria.
12. Vascular disorders: Hypertension.
13. Azelastine hydrochloride nasal spray

The incidence of adverse events in using this product is low (< 2%), including drowsiness, nasal dryness, dry mouth, vivid dreams, abdominal pain, dizziness, cough, and facial flushing, etc. Improper use (such as tilting the head back) may result in symptoms like a pungent taste, nausea, and vomiting.

1. Fluticasone propionate nasal spray

Adverse events include:

1. Very common: Epistaxis.
2. Common: An unpleasant taste and smell after use, headache, nasal and throat dryness, irritation, etc.
3. Very rare: Allergic/anaphylactoid reactions, bronchospasm, rash, facial or tongue edema, nasal septum perforation, glaucoma, increased intraocular pressure, and cataracts, etc.
4. Loratadine tablets

In previous controlled clinical trials, no significant sedative effects have been observed at a recommended dose of 10mg per day.

1. Common: Fatigue, headache, drowsiness, dry mouth, gastrointestinal discomfort including nausea, gastritis, and rash, etc.
2. Rare: Hair loss, allergic reactions, liver function abnormalities, tachycardia, and palpitations, etc.
3. Other: Blurred vision, blood pressure decreases or increase, syncope, hyperkinesia, jaundice, hepatitis, liver necrosis, epileptic seizures, breast enlargement, erythema multiforme, etc.
4. Other adverse events.

**Adverse events recording and management**

An adverse event refers to an unexpected medical occurrence in a clinical trial that may not necessarily have a causal relationship with the clinical trial. Therefore, an adverse event could be a laboratory abnormality or disease symptom caused by the trial product, or it might be unrelated to the trial product. During the trial, adverse events should be recorded truthfully, including the occurrence time, severity, duration, measures taken, and outcomes.

1. Causality between adverse events and treatment is categorized into four grades: definitely related, possibly related, possibly unrelated, and unrelated.
2. Severity assessment criteria for adverse events follow the WHO grading scale for drug reactions. If an adverse reaction not listed in the table occurs, it can be described as follows:
3. Mild: Does not affect the normal functioning of the subject.
4. Moderate: Partially affects the normal functioning of the subject.
5. Severe: Significantly affects the normal functioning of the subject.
6. Management of adverse events:

During the experimental phase, if an adverse event deemed related to the study drug by the investigator (assessed as definitely related or possibly related) occurs, symptomatic treatment should be provided. If the symptoms subside within two days, the treatment is continued as per the original protocol. If the symptoms do not subside, the drug is discontinued immediately and the treatment is resumed once the symptoms have alleviated. Should the symptoms persist without relief within a week, the subject should be withdrawn from the study. In cases of discomfort related to the use of hypertonic seawater nasal irrigation solution, it switches to physiological saline nasal irrigation solution. If there is no improvement, seawater nasal irrigation solution is discontinued, and physiological saline nasal irrigation solution is resumed in conjunction with other treatments once the related symptoms have eased.

**Serious adverse events**

An adverse event is considered serious when it occurs at any drug dose and:

1. Results in death.
2. Is life-threatening.
3. Requires hospitalization or long hospital stay.
4. Results in persistent or significant disability/incapacity.
5. Causes congenital anomalies/birth defects or other medical events.
